# Multiple Organ Dysfunction in Older Major Trauma Critical Care Patients: A Multicenter Prospective Observational Study

**DOI:** 10.1097/AS9.0000000000000174

**Published:** 2022-06-16

**Authors:** Elaine Cole, Chris Aylwin, Robert Christie, Bebhinn Dillane, Helen Farrah, Phillip Hopkins, Chris Ryan, Adam Woodgate, Karim Brohi

**Affiliations:** From the * Centre for Trauma Sciences, Blizard Institute, Queen Mary University, London, United Kingdom; † Imperial College Healthcare NHS Trust, London, United Kingdom; ‡ Barts Health NHS Trust, London, United Kingdom; § St Georges University Hospital NHS Trust, London, United Kingdom; ‖ King’s College Hospital NHS Foundation Trust, London, United Kingdom.

**Keywords:** MODS, multiple organ dysfunction, geriatric, older trauma, frailty, TBI

## Abstract

The objective was to explore the characteristics and outcomes of multiple organ dysfunction syndrome (MODS) in older trauma patients.

**Background:**

Severely injured older people present an increasing challenge for trauma systems. Recovery for those who require critical care may be complicated by MODS. In older trauma patients, MODS may not be predictable based on chronological age alone and factors associated with its development and resolution are unclear.

**Methods:**

Consecutive adult patients (aged ≥16 years) admitted to 4 level 1 major trauma center critical care units were enrolled and reviewed daily until discharge or death. MODS was defined by a daily total sequential organ failure assessment score of >5.

**Results:**

One thousand three hundred sixteen patients were enrolled over 18 months and one-third (434) were aged ≥65 years. Incidence of MODS was high for both age groups (<65 years: 64%, ≥65 years: 70%). There were few differences in severity, patterns, and duration of MODS between cohorts, except for older traumatic brain injury (TBI) patients who experienced a prolonged course of MODS recovery (TBI: 9 days vs no TBI: 5 days, *P* < 0.01). Frailty rather than chronological age had a strong association with MODS development (odds ratio [OR], 6.9; 95% confidence intervals [CI], 3.0–12.4; *P* < 0.001) and MODS mortality (OR, 2.1; 95% CI, 1.31–3.38; *P* = 0.02). Critical care resource utilization was not increased in older patients, but MODS had a substantial impact on mortality (<65 years: 17%; ≥65 years: 28%). The majority of older patients who did not develop MODS survived and had favorable discharge outcomes (home discharge ≥65 years NoMODS: 50% vs MODS: 15%; *P* < 0.01).

**Conclusions:**

Frailty rather than chronological age appears to drive MODS development, recovery, and outcome in older cohorts. Early identification of frailty after trauma may help to predict MODS and plan care in older trauma.

## INTRODUCTION

Managing severely injured older people is an increasing challenge for trauma systems^1,2^ and for many older trauma patients who require critical care admission outcomes are poor.^3,4^ Recovery in this group is complicated by multiple organ dysfunction syndrome (MODS), which is associated with increased mortality and prolonged hospital stay.^5,6^ Previously, older age has been attributed to the development of trauma-related organ dysfunction,^7–9^ but more recently, predictors of MODS after trauma have not included age.^10,11^ As early trauma management has evolved and greater numbers of older people are surviving their initial injuries, factors other than age may contribute to the development, patterns, and resolution of MODS in this group.

Aging is associated with immunosenescence and a proinflammatory state,^12,13^ and these dysfunctional responses may increase vulnerability to MODS.^14^ Recent studies of persistent immunosuppression and prolonged organ dysfunction after trauma have associated older age with poor outcome.^15,16^ Yet physiological deterioration associated with poor outcome such as MODS may not be consistently predictable based on chronological age, where some older people are physiologically healthy preinjury, while others are vulnerable. Preinjury frailty impairs physiological function, independently predicts in-hospital mortality in trauma patients,^17^ and may also be a determinant of MODS development.^18,19^ Specific injuries common to older people may also predispose this group to organ dysfunction, such as traumatic brain injury (TBI), which has a higher morbidity and mortality and greater resource use in older patients compared with younger counterparts.^20,21^ The contemporary factors associated with the development of and recovery from MODS in older major trauma patients are not known.

As the aging population grows and the demand for critical care resource increases, a greater understanding of the predictors and patterns of MODS in older trauma patients will help to stratify those most at risk and develop targeted interventions. The primary aim of the study was to identify the characteristics of older major trauma patients who develop MODS, compared with younger controls. The secondary aim was to explore age-related differences in patterns of MODS and their resolution. The final aim was to examine clinical outcomes associated with MODS in older trauma patients.

## METHODS

### Study Sites and Participants

Study sites were the 4 major trauma centers (MTC) (level 1 equivalent hospitals) within the London Major Trauma System, which are designated to manage the most severely injured patients across a geographical region. Over 18 months from February 2017, consecutive adult trauma patients (≥16 years) requiring admission to critical care were enrolled into the study with informed consent from a nominated consultee (clinician or relative) until the patient regained capacity. Patients with significant burns (>20%), burn-only injuries or non-emergency transfers (>4 hours) from other centers were not approached for inclusion in the study. Older patients were those aged ≥65 years, and younger were aged <65 years. To evaluate potential age-specific characteristics and outcomes within older and younger groups we identified four subcohorts of <45 years (young adults), 45–65 years (middle aged), 65–75 years (older adults), and >75 years (older old). Critical care was defined as either an intensive care unit (level 3 care) or a combined unit of intensive care (level 3 care) and high dependency unit (level 2 care). Ethical approval was granted by the Health Research Authority, London, and South East Research Ethics Committee (IRAS 209230).

### Outcomes

The primary outcome was the development of MODS during the critical care admission period. Secondary outcomes were in-hospital mortality, MODS mortality, infection developed in critical care, ventilator use, critical care and hospital length of stay, and home discharge from the MTC.

### Data Collection

Procedures and training for the site teams were provided by the chief investigator and study research nurses via the Multiple Organ Dysfunction in Elderly Trauma (MODET study) webpage.^22^ Data were collected on demographic characteristics, number of comorbidities, frailty, body mass index, injury type and severity, admission physiology and resuscitation, and the need for emergency surgery or interventional radiology in the first 24 hours from injury. Patients of all ages were screened for the presence of preinjury frailty on or near to critical care admission using the 5-point British Geriatric Society “Recognising frailty syndromes” guidance.^23^ Those suspected of preinjury frailty were then formally assessed with the Edmonton Frail Scale once they had regained capacity.^24^ For patients who remained physically or cognitively impaired, close family or care records were queried to ascertain preinjury cognition status, similar to the process described in other acute settings.^25^ Research teams reviewed patients daily in critical care until discharge or death.

### Definitions

The presence and evolution of MODS were determined with Sequential Organ Failure Assessment (SOFA) scoring.^26,27^ SOFA scores were measured daily from admission as described in earlier critical care studies.^28,29^ MODS was defined as a total SOFA score 6 or more in a 24-hour period.^9,30^ Recovery was when the SOFA score fell and remained below 6 for more than 24 hours. Severe TBI was classified as a head abbreviated injury score (AIS) of greater than 3. Frailty was defined as a score of 8 or more on the Edmonton Frail Scale.^24^ Infection was diagnosed with clinical evidence of infection (eg, observation of purulent exudate, pulmonary in-filtrates on chest radiography) and a positive culture, or requiring treatment with antibiotics.^31^ End of life care was defined as those patients being transferred to supportive, physical, psychological, and spiritual comfort measures.

### Data Analysis

Data were analyzed using Graphpad PRISM (version 9.0.1) and IBM SPSS statistics (version 26). According to Shapiro-Wilk tests, continuous data were nonparametric and therefore compared with Mann-Whitney *U* tests. Categorical variables were analyzed using Chi-squared or Fisher’s Exact tests. Univariate and multivariate logistic regression analysis was used to evaluate the strongest associations for MODS development and MODS mortality in older and younger patients. Variables significant at *P* < 0.10 level in univariate analysis were entered into the multivariate models. Model discrimination and fit were assessed using area under receiver operator curve analysis (reporting AUC and 95% confidence intervals [CI]), and the Hosmer and Lemeshow test. Results are presented as odds ratios (OR) with 95% CI. A *P* value of <0.05 was considered significant. Kaplan-Meier curves were plotted for 30-day MODS survival and age group curves were compared using Log-Rank (Mantel-Cox) tests.

## RESULTS

During the 18-month study period, 1435 consultees and patients were approached for enrollment. Seventy declined to participate, 10 patients died within 24 hours and 39 were transferred for end of life care within 72 hours of admission (of which 32 [82%] were aged ≥65 years). A total of 1316 patients were consented into the study, of which one-third (434) were 65 years old or older (Table 1). Overall, older patients aged 65 years or more were more likely to be female (39% vs 19%), to have sustained a blunt injury (98% vs 84%), and were more likely to be frail (49% vs 5%). The incidence of TBI was similar between groups (≥65 years: 40% vs <65 years: 43%), but the severity was lower for older patients (head AIS, ≥65 years: 4 [3–5] vs <65 years: 5 [4–5], *P* < 0.001). Overall mortality was 17%, but higher in the older group (≥65 years: 28% vs <65 years: 12%) (Table 1).

**TABLE 1.** Admission and Injury Characteristics: All Patients, <65 and ≥65 Year Groups

		<65 y (882)		≥65 y (434)	
	All Patients	No MODS	MODS	No MODS	MODS
	1316	319 (36%)	563 (64%)	137 (30%)	297 (70%)
Age	52 (32–70)	36 (23–50)	40 (28–53)*	80 (73–85)	76 (70–83)^†^
Male (%)	971 (74)	246 (77)	462 (82)	73 (53)	190 (64)
Comorbidities	4 (4–4)	3 (3–4)	3 (2–3)	7 (6–7)	6 (6–7)
Frailty (%)	258 (20)	13 (4)	32 (6)	49 (36)	165 (56)^†^
BMI	24.6 (22.6–27.4)	24.6 (22.6–28.1)	24.6 (22.6–27.1)	24.6 (22.2–27.6)	25.7 (23.4–28.3)^†^
Blunt injury (%)	1166 (89)	252 (79)	490 (87)^†^	133 (97)	291 (98)
TBI (%)	557 (42)	81 (25)	302 (54)*	44 (32)	130 (44)^†^
ISS	25 (16–33)	21 (13–29)	27 (24–38)*	18 (9–25)	25 (14–29)*
Head AIS	4 (3–5)	4 (3–5)	5 (4–5)*	3 (3–4)	4 (3–5)*
Thoracic AIS	3 (3–4)	3 (3–4)	3 (3–4)	3 (3–4)	3 (3–4)
Abdominal AIS	3 (2–4)	3 (2–4)	3 (2–4)	3 (2–3)	3 (2–3)
Extremity AIS	2 (2–3)	2 (2–3)	2 (2–3)	2 (2–3)	2 (2–3)
First SBP	130 (106–150)	125 (103–140)	126 (104–147)	140 (123–163)	137 (107–160)
First GCS	13 (7–15)	15 (12–15)	9 (4–14)*	15 (13–15)	14 (8–15)*
Admission BD mEq/L	3.3 (0.7–6.3)	2.9 (0.3–5.6)	4.5 (2.2–8.7)*	1.9 (–0.2 to 3.5)	2.9 (0.3–5.8)*
Admission INR	1.1 (1.0–1.2)	1.1 (1.0–1.1)	1.1 (1.0–1.2)	1.1 (1.0–1.2)	1.1 (1.0–1.3)
CSL (L)/24 h	2.1 (1.1–3.4)	2.0 (1.0–3.5)	2.4 (1.4–3.8)^†^	1.4 (1.0–2.4)	2.0 (1.0–3.0)^†^
RBC units/24 h	4 (2–7)	4 (2–6)	5 (3–7)^†^	2 (2–4)	3 (2–5)
FFP/24 h	4 (3–6)	4 (2–6)	4 (3–6)	2 (2–4)	4 (2–5)
Platelets/24 h	1 (1–2)	1 (1–2)	1 (1–2)	1 (1–1)	1 (1–2)
Surgery/IR <24 h (%)	327 (25)	87 (27)	181 (32)	10 (7)	49 (16)*

Data presented as median (IQR) or n (%). Frail status unknown – All patients: 10, No MODs ≥65: 4, MODS ≥65: 6.

*P < 0.001 comparing MODS and No MODS groups (Mann-Whitney *U* test, Chi-squared, or Fishers Exact test).

^†^*P* < 0.05.

AIS indicates Abbreviated Injury Score; BD, base deficit; BMI, body mass index; CSL, crystalloid; FFP, fresh frozen plasma; GCS, Glasgow Coma Scale; INR, international normalized ratio; IR, interventional radiology; ISS, Injury Severity Score; RBC, red blood cells; SBP, systolic blood pressure; TBI, traumatic brain injury.

There was no difference in MODS incidence between older patients (70%) and those under 65 years (64%) (Table 1) and across the 4 age sub-cohorts (Supplemental Table 1, http://links.lww.com/AOSO/A131). Irrespective of age, the younger MODS patients (<45 years and 45–65 years) had similar rates of TBI, were more severely injured, shocked on arrival, and received more fluids (Supplemental Table 1, http://links.lww.com/AOSO/A131). There were few other characteristic differences in older subcohorts (65–75 years and >75 years) except for significantly increased rates of frailty in those who developed MODS (Supplemental Table 1, http://links.lww.com/AOSO/A131). Factors associated with the development of MODS were injury severity, admission shock, and crystalloid volume administered within the first 24 hours for both older and younger patients (Table 2). Frailty was strongly associated with the development of MODS in older patients (adjusted OR 6.9; 95% CI 3.0–12.4; *P* < 0.001), but not TBI.

**TABLE 2.** Factors Associated With the Development of MODS in Younger and Older Cohorts

	Univariate Analysis <65 yOdds Ratio (95% CI), *P*	Multivariate Analysis <65 yOdds Ratio (95% CI), *P*	Univariate Analysis ≥65 yOdds Ratio (95% CI), *P*	Multivariate Analysis ≥65 yOdds Ratio (95% CI), *P*
Age	1.02 (1.01–1.03), <0.01	1.02 (1.00–1.04), 0.05	0.96 (0.93–0.98), <0.01	**0.92 (0.88–0.97), <0.01**
Male	1.34 (0.96–1.88), 0.08	1.43 (0.59–3.50), 0.423	0.64 (0.42–0.96), 0.04	0.74 (0.37–1.46), 0.388
Comorbidities	0.96 (0.88–1.01), 0.165	–	0.85 (0.78–0.99), 0.04	0.82 (0.68–1.08), 0.08
Frailty	1.44 (0.75–2.79), 0.271	–	2.24 (1.47–4.22), <0.001	**6.96 (3.0–12.4), <0.001**
BMI	0.96 (0.94–0.98), 0.03	0.95 (0.90–1.03), 0.05	1.05 (1.008–1.11), 0.03	1.08 (0.98–1.18), 0.06
Blunt injury	0.70 (0.44–1.11), 0.155	–	1.26 (1.00–2.88), 0.05	0.34 (0.95–1.22), 0.99
TBI	3.40 (2.52–4.60), <0.001	**2.41 (1.60–3.62), <0.001**	1.64 (1.07–2.51), 0.02	1.80 (0.80–4.05), 0.150
ISS	1.06 (1.04–1.07), <0.001	**1.05 (1.03–1.07), <0.001**	1.04 (1.02–1.06), <0.001	**1.08 (1.04–1.12), <0.001**
First SBP	1.04 (0.99–1.09), 0.146	–	0.99 (0.85–1.11), 0.151	–
First GCS	0.82 (0.79–0.95), <0.001	–	0.90 (0.85–0.96), <0.01	–
Admission BD mEq/L	1.06 (1.03–1.09), <0.001	**1.07 (1.03–1.10), <0.01**	1.03 (1.05–1.21), <0.01	**1.11 (1.09–1.23), 0.04**
Admission INR	1.74 (0.89–3.39), 0.104	–	1.25 (0.92–1.70), 0.139	–
CSL (L)/24 h	1.10 (1.02–1.19), <0.01	**1.11 (1.01–1.21), 0.01**	1.21 (1.01–1.45), 0.02	**1.50 (1.2–2.07), <0.001**
RBC units/24H	1.06 (1.009–1.12), 0.03	1.07 (0.98–1.16), 0.131	1.14 (0.92–1.42), 0.205	–
FFP/24H	1.005 (0.97–1.04), 0.769	–	1.17 (0.77–1.57), 0.277	–
Platelets/24H	1.14 (0.92–1.41), 0.219	–	3.09 (0.50–13.8), 0.912	–
Surgery/IR <24H	1.59 (1.14–2.21), 0.01	1.32 (0.63–2.75), 0.455	1.02 (0.69–1.66), 0.932	–

Data presented as Odds Ratio (95% CI), *P* value. Bold font indicates *P* value <0.05.

Variables less than 0.1 in univariate analysis entered into the multivariable model. GCS collinear with TBI therefore omitted from models. Under 65 y Hosmer and Lemeshow test: 4.945, p=0.763, AUC 0.79 (95% CI: 0.74-0.86, p<0.001). Over 65y Hosmer and Lemeshow test: 9.257, p=0.296, AUC 0.82 (95% CI: 0.76-0.87, p<0.01) (ROC curve analysis in Supplemental Figure 3, http://links.lww.com/AOSO/A131).

BD, base deficit; BMI, body mass index; CSL, crystalloid; FFP, fresh frozen plasma; GCS, Glasgow Coma Scale; INR, international normalized ratio; IR, interventional radiology; ISS, Injury Severity Score; RBC, red blood cells; SBP, systolic blood pressure; TBI, traumatic brain injury.

**FIGURE. 1.**
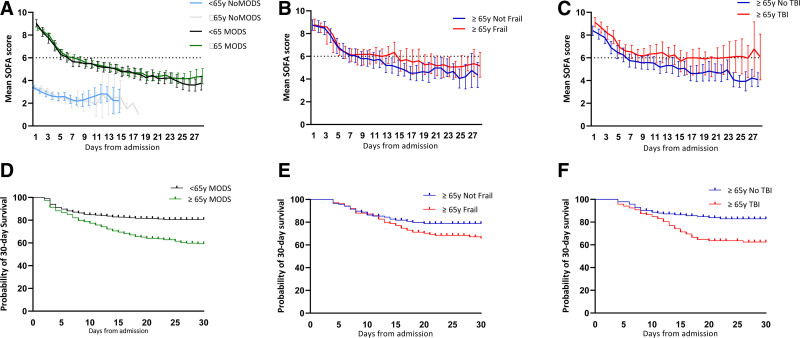
A, Mean (95% CI) SOFA scores by day of hospital admission: all patients, with and without MODS. B, Mean (95% CI) SOFA scores by day of hospital admission: patients aged ≥65 years with and without preinjury frailty. C, Mean (95% CI) SOFA scores by day of hospital admission: patients aged ≥65 years with and without TBI. D, Kaplan-Meier 30-day survival curve for patients with MODS: Log-rank (Mantel-Cox) test 37.0, *P* < 0.001. E, Kaplan-Meier 30-day survival curve for patients with MODS aged ≥65 years with and without preinjury frailty: Log-rank (Mantel-Cox) test 4.72, *P* = 0.03. F, Kaplan-Meier 30-day survival curve for patients with MODS aged ≥65 years with and without TBI: Log-rank (Mantel-Cox) test 10.6, *P* < 0.001. CI, confidence intervals; MODS, multiple organ dysfunction syndrome; SOFA, sequential organ failure assessment; TBI, traumatic brain injury.

Overall there was no difference in the initial severity of MODS between older and younger patients (max SOFA, ≥65 years: 9.1 vs <65 years: 9.9; day of max SOFA, ≥65 years: day 1 vs <65 years: day 1; Fig. 1A). Respiratory, cardiovascular, and central nervous system (CNS) dysfunction were the greatest contributors to MODS in older patients (Supplemental Figures 1 and 2, http://links.lww.com/AOSO/A131) and overall, proportions affected were similar for younger patients (respiratory dysfunction: older 94%, younger 89%; cardiovascular dysfunction: older 79%, younger 74%; CNS dysfunction: older 76%, younger 70%). Secondary infection rates were the same in younger and older MODS patients (Table 3). There was no overall difference in the time to MODS recovery in survivors (≥65 years: 7 days vs <65 years: 6 days) (Fig. 1A). On average older MODS patients spent less time on a ventilator and less time in critical care compared with younger MODS patients (Table 3; Supplemental Table 2, http://links.lww.com/AOSO/A131). Although frailty was associated with the development of MODS in older patients, it was not related to the time to MODS recovery (Fig. 1B). TBI in older patients was associated with a more prolonged course of MODS, where resolution took on average 9 days, compared with 5 days in the no TBI group (*P* < 0.01; Fig. 1C), and this was driven principally by the central nervous system component of the SOFA score (Supplemental Figure 2, http://links.lww.com/AOSO/A131).

**TABLE 3.** Outcomes: All Patients, <65 and ≥65 Year Groups

		<65 y (882)		≥65 y (434)	
	All Patients	No MODS	MODS	No MODS	MODS
	319 (36%)	563 (64%)	137 (30%)	297 (70%)	1316
Days to MODS recovery~	6 (3–11)	–	6 (3–12)	–	7 (2–10)*
Ventilator days~	6 (2–13)	1 (1–2)	11 (5–16)	1 (1–2)	8 (3–17)*
CCLOS~	8 (4–17)	4 (2–6)	16 (9–24)	4 (3–7)	12 (7–22)*
Infection in critical care (%)	335 (25)	23 (7)	190 (34)	21 (15)	101 (34)
HLOS~	26 (14–43)	13 (8–24)	37 (23–57)	18 (13–29)	30 (20–44)*
Mortality (%)	227 (17)	0	107 (19)	14 (10)	106 (36)*
Died in critical care (%)	186 (14)	0	96 (17)	7 (5)	83 (28)*
Died EoL >72 h (%)	50 (4)	0	10 (2)	7 (5)	33 (11)*
Home discharge from MTC (%)	540 (41)	245 (77)	181 (32)	68 (50)	46 (15)*

Data presented as median (IQR) unless otherwise indicated.

*P < 0.05 for comparison between <65 y and ≥65 y MODS groups (Mann-Whitney U test, Chi-squared or Fishers Exact test).

CCLOS indicates critical care length of stay; HLOS, hospital length of stay; EoL, end of life care; MTC, major trauma center; ~, survivors only.

Mortality was worse for patients who developed MODS compared with no MODS cohorts, irrespective of age (Table 3; Supplemental Table 2, http://links.lww.com/AOSO/A131). Comparing older and younger patients, mortality was almost twice as high in those with MODS aged 65 or more years compared with younger (≥65 years: 36% vs <65 years: 19%, *P* < 0.001; Fig. 1D) and more than 3 times greater than when comparing older patients with MODS to older patients without MODS (36% vs 10%, *P* < 0.001). Over a quarter of older MODS patients died in critical care (older: 28% vs younger: 17%, *P* < 0.001), and this rose to a third in the oldest old group (>75 years: 34%, Table 3). MODS mortality increased further in frail older patients (41% vs 28%, *P* = 0.02), and in those with TBI (42% vs 31%, *P* = 0.03) but later deaths following end of life care were not greater in either cohort (Fig. 1E,F; Supplemental Table 3, http://links.lww.com/AOSO/A131). In multivariate analysis, the presence of frailty, TBI and “younger” older age were associated with mortality in the older MODS patients (Table 4). Older survivors who had developed MODS were less likely to be discharged directly to their own home than younger patients (15% vs 32%, *P* < 0.001; Table 3; Supplemental Table 2, http://links.lww.com/AOSO/A131), whereas half of older patients who did not develop MODS were discharged to their usual place of residence. There were no differences in home discharge from the MTC for MODS patients who were frail or with a TBI, although rates were very low in both groups (Supplemental Table 3, http://links.lww.com/AOSO/A131).

**TABLE 4.** Factors Associated With MODS Mortality in Younger and Older Cohorts

	Univariate Analysis <65 yOdds Ratio (95% CI), *P*	Multivariate Analysis <65 yOdds Ratio (95% CI), *P*	Univariate Analysis ≥65 yOdds Ratio (95% CI), *P*	Multivariate Analysis ≥65 yOdds Ratio (95% CI), *P*
Age	0.98 (0.96–0.99), 0.03	**0.97 (0.95–0.99), <0.01**	0.96 (0.93–0.99), 0.02	**0.96 (0.93–0.99), 0.01**
Male	1.28 (0.76–2.13), 0.345	–	0.92 (0.56–1.52), 0.764	–
Comorbidities	0.95 (0.68–1.55), 0.364	–	1.02 (0.91–1.14), 0.720	–
Frailty	0.92 (0.38–2.17), 0.898	–	1.77 (1.08–2.92), 0.02	**2.10 (1.32–3.38), 0.02**
BMI	1.01 (0.96–1.07), 0.499	–	1.01 (0.96–1.16), 0.630	–
Blunt injury	0.97 (0.52–1.92), 0.935	–	1.01 (0.50–2.03), 0.978	–
TBI	2.12 (1.37–3.28), <0.001	**3.0 (1.8–5.1), <0.001**	1.57 (1.16–3.22), 0.01	**2.05 (1.30–3.24), 0.02**
ISS	1.07 (1.05–1.09), 0.02	**1.08 (1.06–1.09), 0.04**	1.00 (0.98–1.02), 0.959	–
First SBP	0.99 (0.94–1.01), 0.102	–	0.99 (0.98–1.04), 0.460	–
First GCS	0.91 (0.83–1.06), 0.794	–	1.17 (1.01–1.25), 0.01	–
Admission BD mEq/L	1.92 (1.89–1.95), <0.001	**1.8 (1.7–1.9), 0.01**	0.94 (0.90–1.00), 0.05	0.95 (0.89–1.009), 0.101
Admission INR	1.03 (0.51–2.08), 0.989	–	0.99 (0.26–1.25), 0.938	–
CSL (L)/24 h	0.83 (0.73–1.19), 0.204	–	0.98 (0.88–1.17), 0.902	–
RBC units/24 h	0.94 (0.91–0.98), 0.04	0.94 (0.93–1.00), 0.05	0.96 (0.89–1.19), 0.579	–
FFP/24 h	0.99 (0.95–1.04), 0.964	–	0.99 (0.84–1.01), 0.902	–
Platelets/24 h	1.003 (0.86–1.06), 0.967	–	0.55 (0.26–1.18), 0.127	–
Surgery/IR <24 h	0.88 (0.56–1.39), 0.601	–	0.86 (0.54–1.70), 0.901	–

Data presented as Odds Ratio (95% CI), *P* value. Bold font indicates *P* value <0.05.

Variables less than 0.1 in univariate analysis entered into the multivariable model. GCS collinear with TBI therefore omitted from models. Under 65 y Hosmer and Lemeshow test: 4.609, *P* = 0.798, AUC 0.75 (95% CI, 0.69–0.80, *P* < 0.001). Over 65 y Hosmer and Lemeshow test: 12.51, *P* = 0.130, AUC 0.68 (95% CI, 0.61–0.72, *P* < 0.01) (ROC curve analysis in Supplemental Figure 3, http://links.lww.com/AOSO/A131).

BD indicates base deficit; BMI, body mass index; CSL, crystalloid; FFP, fresh frozen plasma; GCS, Glasgow Coma Scale; INR, international normalized ratio; IR, interventional radiology; ISS, Injury Severity Score; RBC, red blood cells; SBP, systolic blood pressure; TBI, traumatic brain injury.

## DISCUSSION

This was prospective multicentre study in major trauma critical care patients, which compared MODS in older and younger populations. Overall the incidence of MODS was high across age groups and there were few differences in the severity, patterns and duration of MODS. Rates of females and TBI were increased in older patients, neither of which were associated with MODS development. Resource utilization was not increased in the older group, but MODS had a substantial impact on survival. Frailty, rather than chronological age, had a strong association with the development of MODS and subsequent outcomes. The majority of older patients who did not develop MODS survived and had favorable discharge outcomes, even in the “oldest old” group.

Overall, the severity and patterns of MODS were very similar between age cohorts and organ dysfunction was at its most severe on the first day of critical care for older and younger patients. There was no strong signal of the persistent inflammation and organ dysfunction reported in other older trauma populations,^16,32^ where critical care stays exceed 14 days and are characterized by immunocompromise with recurrent nosocomial infections.^33,34^ This was not the case in our older MODS patients and critical care resource use was not significantly increased in comparison to younger people. Organ dysfunction recovery trajectories only differed in the presence of TBI, where the older group had a more indolent course.

Preinjury frailty was a significant burden for our older patients, and frailty rather than older age was strongly associated with the development of MODS, independent of TBI. Frailty is characterized by decline in physiological and cognitive systems, which results in vulnerability to stressors such as major traumatic injury.^35,36^ Frailty is known to be prognostic of adverse events in general and critical care older trauma populations^37,38^ and frail-specialist pathways such as early screening and geriatric consults have resulted in improved outcomes.^39^ The strong association we found between frailty and MODS development suggests that timely identification of frailty in critical care may predict likely outcomes and help to stratify those who require frail-specific management early in the clinical course.

Overall, more than a third of the older patients with MODS died and this is considerably higher than other older critical care trauma populations,^4,38,40^ albeit these studies had lower average injury severity (<15) than our cohort. Although higher unadjusted death rates were observed in the very old, frailty, and TBI but not increased “older age,” were strong determinants of mortality in our older MODS group. Other outcomes were worse for older patients in the presence of MODS, where this cohort were less likely to be discharged to home in comparison to younger counterparts. However for our older patients who did not develop MODS, outcomes were significantly better and the majority survived. Overall half of the older group were discharged directly to home, plus 42% of the oldest patients, both of which compare favorably to other large studies of geriatric cohorts within similar trauma systems where home discharge rates were 23%^41^ and 36%.^42^

Older MODS patients with TBI had a protracted pattern of resolution, which is likely to be attributed to the time to resolution of the CNS component of the SOFA score. TBI has been associated with “prolonged MODS” in recent studies but unlike our patients, not specifically in older age.^43,44^ A complex interaction between systemic inflammation and CNS dysfunction has been linked to MODS development in adult TBI patients^45,46^; however, this has not been examined in older polytrauma cohorts. TBI was strongly associated with mortality in all ages, with the greatest proportion of deaths in older MODS patients and significantly higher than other studies. Although advanced age is reported to be predictive of in-hospital mortality for patients with brain injuries,^47,48^ deaths associated with TBI *and* MODS, which cannot be attributed to withdrawal of care, remain a significant burden in trauma critical care.

## LIMITATIONS

There are a number of limitations to this study. SOFA scoring differs from other MODS scores such as Denver,^49^ as it includes a CNS component, which may partly account for differences in the proportion and trajectory of MODS in this study compared with others. However, the use of SOFA in severely injured populations with TBI has good discriminative ability, and balance of sensitivity and specificity in predicting unfavorable outcome for injured patients.^26,50^ We defined frailty as a binary measure of “yes or no” and we acknowledge that frailty has degrees of severity on a continuum and it may be that those with more severe frailty were most at risk of MODS and poor outcome within this study. We also did not collect in-depth comorbidity detail and this may be linked to frail status. Further large scale exploration of older critical care trauma populations is required with detailed frailty evaluation. Finally, it was not possible to identify complete causes of death in critical care as we did not have access to postmortem reports therefore we were not able to definitively assign causation to MODS with or without the presence of TBI.

## CONCLUSION

In contemporary trauma critical care, MODS remains a burden for all ages; however, frailty and TBI rather than chronological age appear to drive its development, recovery, and outcome in older cohorts. In contrast, older patients who did not develop MODS had reasonable outcomes following critical care trauma admission. Identification of frailty early in critical care may help to predict likely outcomes and plan appropriate management in older trauma patients.

## ACKNOWLEDGMENTS

The NET research team, St Marys Hospital; The ACET research team, Kings College Hospital, The Critical Care research team, St Georges Hospital; Centre for Trauma Sciences research team, Royal London Hospital.

This study was funded by the Dunhill Medical Trust (R460/0216).

## Supplementary Material


