# Supplementary material for: Multiple Organ Dysfunction in Older Major Trauma Critical Care Patients: A Multicenter Prospective Observational Study
Source: Ann Surg Open. 2022 Jun 16;3(2):e174. doi: 10.1097/AS9.0000000000000174 (PMC10013163; doi:10.1097/AS9.0000000000000174)
Supplement: Supplementary file 1 [file as9-3-e174-s001.pdf]

Supplemental Figure 1. Individual organ components of MODS in patients aged ≥65 years with and without pre-injury frailty

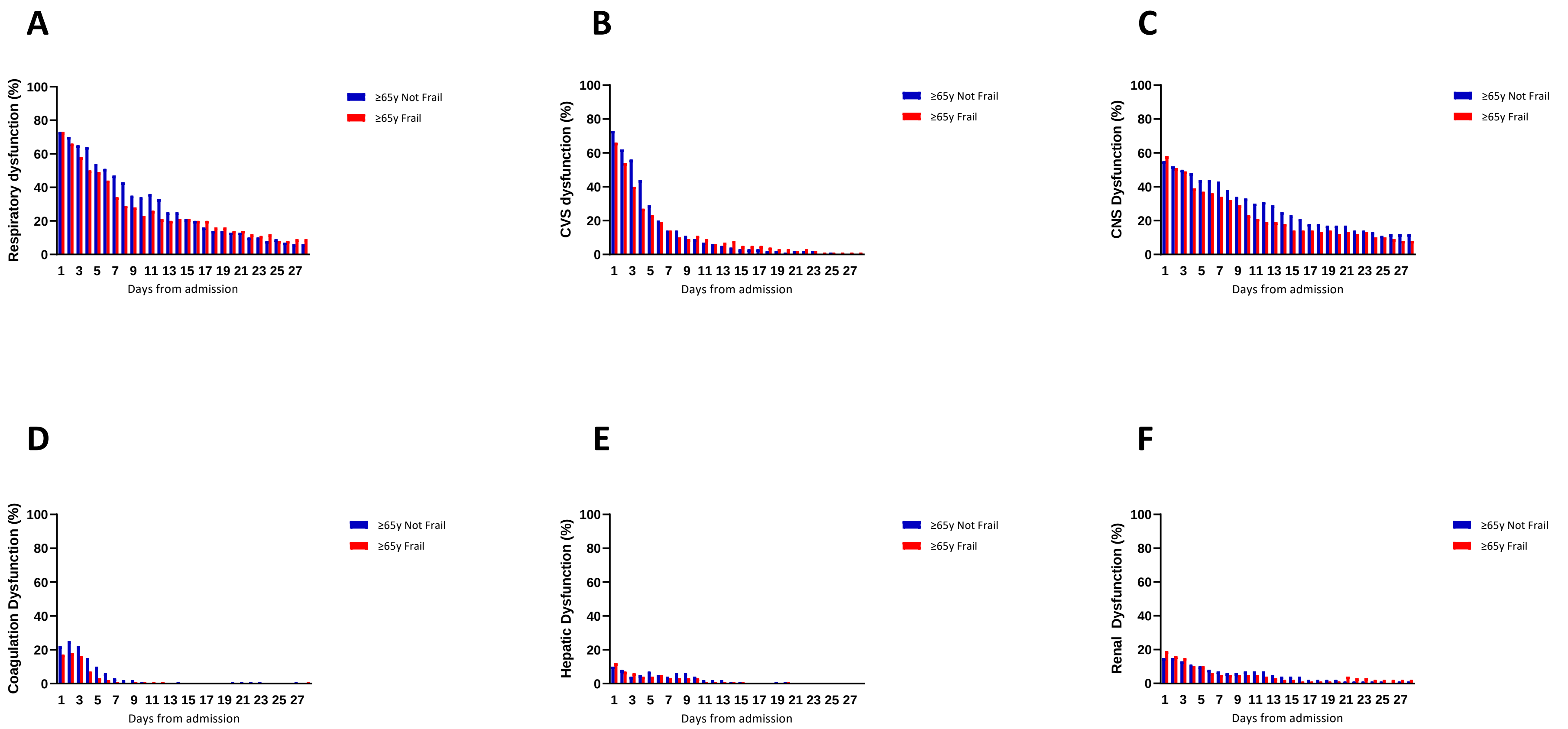

Supplemental figure 1. Individual organ components of MODS in patients aged ≥65 years with and without pre-injury frailty. A. Respiratory **B.** Cardiovascular **C.** Central Nervous System. **D**. Coagulation. **E** Hepatic. **F**. Renal.

Supplemental Figure 2. Individual organ components of MODS in patients aged ≥65 years with and without TBI

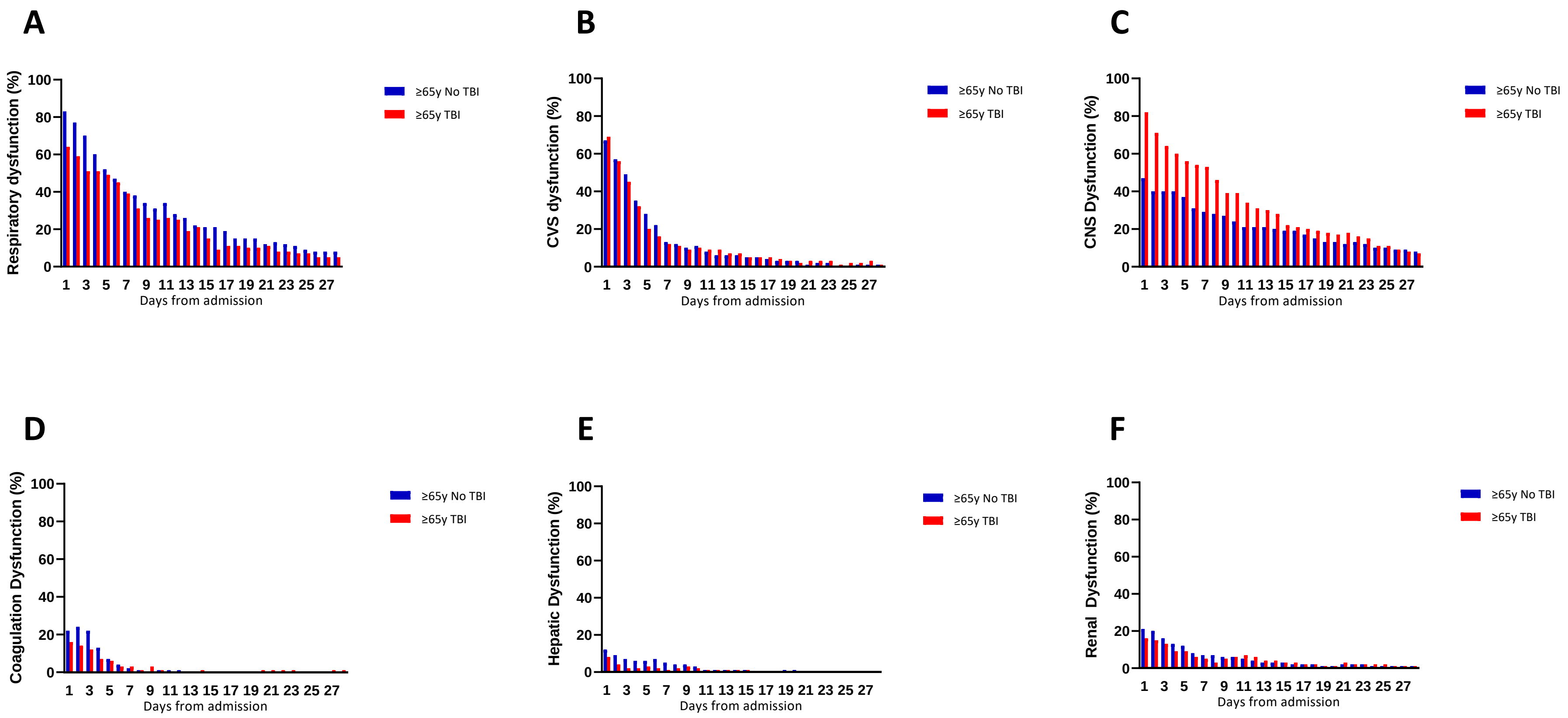

Supplemental figure 2. Individual organ components of MODS in patients aged ≥65 years with and without TBI. A. Respiratory **B.** Cardiovascular **C.** Central Nervous System. **D**. Coagulation. **E** Hepatic. **F**. Renal.

Supplemental Figure 3

A

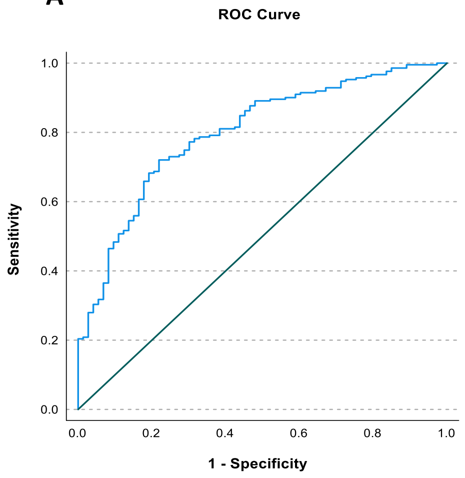

B

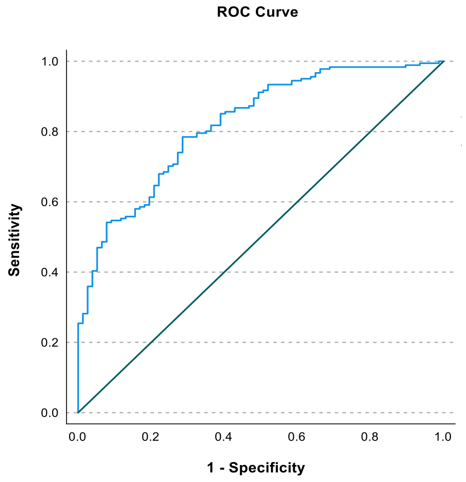

C

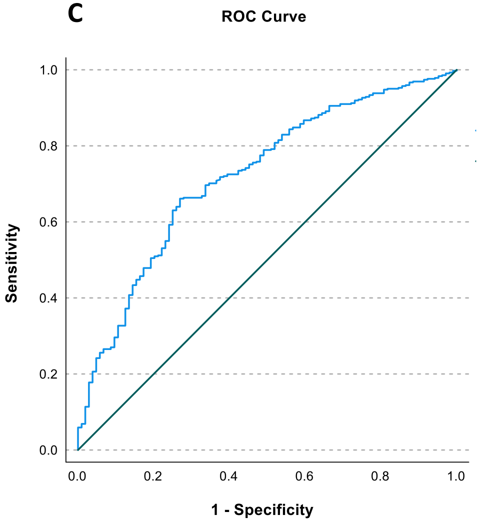

D

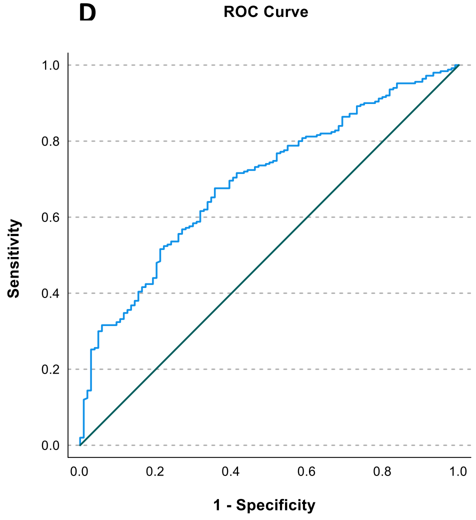

Supplemental figure 3. Receiver operating curves depicted an area under the curve values (AUC) (95% Confidence intervals). A. Factors associated with the development of MODS in <65y AUC 0.79 (95% CI: 0.74-0.86, p<0.001). **B.** Factors associated with the development of MODS in ≥65y AUC 0.82 (95% CI: 0.76-0.87, p<0.01). **C.** Factors associated with MODS mortality in <65y AUC 0.75 (95% CI: 0.69-0.80, p<0.001). **D.** Factors associated with MODS mortality in ≥65y AUC 0.68 (95% CI: 0.61-0.72, p<0.01).

Supplemental Table 1. Admission and injury characteristics

		<45 years		45-64 years		65-75 years		>75 years	
	**All patients**	**No MODS**	**MODS**	**No MODS**	**MODS**	**No MODS**	**MODS**	**No MODS**	**MODS**
	1316	211 (40%)	322 (60%)	108 (31%)	241 (69%)	49 (25%)	146 (75%)	88 (37%)	151 (67%)
**Age**	52 (32-70)	25 (20-30)	26 (21-34)	54 (49-59)	54 (50-59)	71 (69-74)	70 (67-72)	83 (80-87)	82 (80-87)
**Male (%)**	971 (74)	167 (79)	270 (84)	79 (73)	192 (80)	32 (65)	101 (61)	41 (47)	89 (59)
**Co-morbidities**	4 (4-4)	0 (0-4)	0 (0-1)	2 (2-3)	2 (2-3)	5 (4-6)	6 (5-6)	7 (6-7)	6 (5-6)
**Frailty (%)**	258 (20)	2 (1)	4 (1)	11 (10)	27 (11)	7 (14)	63 (45)**	42 (50)	102 (68)*
**BMI**	24.6 (22.6-27.4)	25.9 (22.6-28.1)	24.7 (22.6-27.1)	25.9 (23.4-26.9)	24.8 (23-27.8)	26.1 (23.4-29.4)	26.6 (24-29.3)	24.2 (21.6-27)	25 (22.6-27.7)
**Blunt injury (%)**	1166 (89)	155 (73)	267 (83)*	97 (90)	223 (93)	47 (96)	144 (99)	86 (98)	147 (97)
**TBI (%)**	557 (42)	51 (24)	170 (53)**	30 (28)	132 (55)**	16 (33)	69 (47)	28 (32)	61 (40)
**ISS**	25 (16-33)	20 (13-29)	29 (22-37)**	22 (13-29)	25 (24-38)**	19 (13-25)	25 (17-34)**	17 (9-25)	24 (12-29)*
**Head AIS**	4 (3-5)	4 (3-5)	5 (3-5)**	4 (3-5)	5 (4-5)**	3 (3-4)	4 (3-4)	3 (3-4)	4 (3-4)
**Thoracic AIS**	3 (3-4)	3 (3-4)	3 (3-4)	3 (3-4)	3 (3-4)	3 (3-4)	3 (3-4)	3 (3-4)	3 (3-4)
**Abdominal AIS**	3 (2-4)	3 (2-4)	3 (2-4)	3 (2-4)	3 (2-4)	3 (2-3)	3 (2-3)	3 (2-3)	3 (2-3)
**Extremity AIS**	2 (2-3)	2 (2-3)	2 (2-3)	2 (2-3)	2 (2-3)	2 (2-3)	2 (2-3)	2 (2-2)	2 (2-2)
**First SBP**	130 (106-150)	105 (83-131)	114 (90-132)	132 (108-155)	121 (101-145)	135 (117-158)	137 (112-170)	141 (128-168)	132 (107-155)*
**First GCS**	13 (7-15)	15 (14-15)	9 (4-14)**	15 (11-15)	10 (4-14)**	15 (14-15)	14 (9-15)*	15 (13-15)	14 (7-15)**
**Admission BD mEq/L**	3.3 (0.7-6.3)	3.2 (0.3-6.0)	4.9 (2.1-8.3)**	2.6 (0.4-4.2)	4.3 (1.9-7.4)**	2.7 (1.3-3.8)	3.6 (2.6-4.3)*	1.2 (0.7-1.8)	3.5 (2.8-4.2)**
**Admission INR**	1.1 (1.0-1.2)	1.1 (1.0-1.1)	1.1. (1.0-1.1)	1.1 (1.0-1.1)	1.1. (1.0-1.2)	1.1. (1.0-1.2)	1.1. (1.0-1.3)	1.1. (1.0-1.2)	1.1. (1.0-1.3)
**CSL (L)/24 hours**	2.1 (1.1-3.4)	2.0 (1.0-3.6)	2.9 (1.7-4.1)*	2.0 (1.0-3.4)	2.5 (1.3-3.9)*	1.3 (0.9-1.8)	2.0 (1.7-2.3)*	1.7 (1.3-2.2)	2.1 (1.8-2.4)*
**RBC units/24H**	4 (2-7)	4 (2-5)	4 (3-7)	3 (2-5)	5 (2-7)*	2 (2-3)	3 (2-5)	2 (2-4)	3 (2-5)
**FFP/24H**	4 (3-6)	4 (2-6)	4 (3-6)	4 (2-5)	4 (2-6)	2 (2-4)	3 (2-4)	2 (2-4)	3 (2-5)
**Platelets/24H**	1 (1-2)	1 (1-2)	1 (1-2)	1 (1-2)	1 (1-2)	1 (1-1)	1 (1-2)	1 (1-1)	1 (1-2)
**Surgery/IR <24H (%)**	327 (25)	68 (32)	108 (34)	19 (18)	73 (30)*	3 (6)	22 (15)	7 (8)	27 (18)*

Data presented as median (IQR) or n (%). (%). BMI: Body Mass Index; TBI: Traumatic Brain Injury; ISS: Injury Severity Score; AIS: Abbreviated Injury Score; SBP: Systolic Blood Pressure; GCS: Glasgow Coma Scale; BD: Base Deficit; INR: International Normalised Ratio; CSL: Crystalloid; RBC: Red Blood Cells; FFP: Fresh Frozen Plasma; IR: Interventional Radiology. Frail status unknown – All patients: 10, 65-75y MODS 6, >75y NoMODS: 4. *p<0.05, **p<0.001 comparing NoMODS and MODS groups (Mann Whitney U test, Chi Squared or Fishers Exact test).

Supplemental Table 2. Outcomes

		<45 years		45-64 years		65-75 years		>75 years	
	**All patients**	**No MODS**	**MODS**	**No MODS**	**MODS**	**No MODS**	**MODS**	**No MODS**	**MODS**
**n**	1316	211 (40%)	322 (60%)	108 (31%)	241 (69%)	49 (25%)	146 (75%)	88 (37%)	151 (67%)
**Ventilator days~**	6 (2-13)	1 (1-2)	11 (5-17)**	2 (1-2)	12 (2-19)**	4 (1-8)	8 (5-21)**	2 (1-2)	7 (3-15)**
**CCLOS~**	8 (4-17)	4 (2-5)	15 (9-23)**	4 (2-7)	16 (9-25)**	4 (3-7)	14 (8-25)**	4 (3-7)	9 (5-21)**
**Infection (%)**	335 (25)	12 (6)	105 (33)**	11 (10)	85 (35)**	8 (16)	53 (36)*	13 (15)	48 (44)*
**HLOS~**	26 (14-43)	13 (8-24)	34 (21-53)**	16 (10-25)	39 (27-59)**	18 (12-23)	31 (23-47)**	19 (13-34)	30 (19-40)**
**Mortality (%)**	227 (17)	0	46 (14)*	0	61 (25)**	1 (2)	39 (27)**	13 (15)	67 (44)**
**Died in critical care (%)**	186 (14)	0	46 (14)*	0	50 (21)**	1 (2)	31 (21)**	6 (7)	52 (34)**
**Died EoL >72H (%)**	50 (4)	0	1 (<1)	0	9 (4)	0	6 (4)	7 (8)	27 (18)*
**Home discharge from MTC (%)**	540 (41)	168 (80)	133 (41)**	77 (71)	48 (20)**	31 (63)	32 (22)**	37 (42)	14 (9)**

Data presented as median (IQR) unless otherwise indicated. CCLOS: Critical care length of stay; HLOS: Hospital length of stay; EoL: End of Life care; MTC: Major Trauma Centre*p<0.05, **p<0.001 comparing NoMODS and MODS groups (Mann Whitney U test, Chi Squared or Fishers Exact test).

Supplemental Table 3. Characteristics of MODS in Frail and TBI ≥65 year groups

	**MODS ≥65 years Not Frail**	**MODS ≥65 years Frail***		**MODS ≥65 years No TBI**	**MODS ≥65 years TBI**	
n	**126**	**165**	p value	**167**	**130**	p value
Characteristics						
**Age**	72 (68-77)	79 (72-85)	<0.001	78 (70-84)	75 (69-82)	0.05
**Male (%)**	86 (68)	100 (61)	0.21	113 (68)	77 (59)	0.64
**Co-morbidities**	4 (4-4)	5 (4-5)	0.17	4 (4-5)	4 (2-5)	0.03
**Frail (%)**	n/a	n/a	-	99 (59)	66 (51)	0.37
**BMI**	26 (24-29)	25 (23-28)	0.26	26.0 (23.5-29.0)	25.0 (23.1-27.9)	0.07
**Blunt (%)**	122 (97)	163 (99)	0.40	162 (97)	129 (99)	0.22
**TBI (%)**	59 (47)	66 (40)	0.28	n/a	n/a	-
**ISS**	25 (17-34)	22 (13-27)	<0.001	17 (9-24)	26 (25-38)	<0.001
**First SBP**	130 (100-163)	137 (112-161)	0.34	122 (94-146)	150 (128-173)	<0.001
**First GCS**	14 (11-15)	14 (13-15)	0.89	15 (11-15)	10 (6-14)	<0.001
**Admission BD mEq/L**	2.8 (0.4-5.5)	2.8 (0.1-5.0)	0.83	2.8 (0.3-5.7)	3.0 (0.7-5.9)	0.76
**CSL (L)/24 hours**	1.0 (1.0-3.2)	1.5 (1.0-2.8)	0.05	2.0 (1.0-3.2)	1.8 (1.0-3.0)	0.36
**RBC units**	4 (2-6)	2 (1-5)	0.02	4 (2-6)	3 (2-5)	0.31
**Surgery/IR <24H (%)**	19 (15)	29 (18)	0.63	20 (12)	29 (22)	0.02
Outcomes						
**Days to MODS recovery~**	6 (5-7)	8 (6-9)	0.12	6 (5-7)	7 (6-8)	0.59
**Ventilator days~**	8 (5-17)	8 (3-16)	0.25	7 (3-17)	9 (5-16)	0.22
**CCLOS~**	14 (8-23)	10 (6-22)	0.01	11 (6-22)	13 (7-23)	0.76
**Infection in critical care (%)**	50 (40)	51 (31)	0.18	60 (36)	42 (28)	0.33
**HLOS~**	35 (23-47)	28 (18-37)	0.01	30 (20-42)	32 (20-47)	0.51
**Mortality (%)**	35 (28)	67 (41)	0.02	51 (31)	55 (42)	0.03
**Died in critical care (%)**	30 (24)	49 (30)	0.28	40 (24)	43 (33)	0.09
**Died EoL >72H (%)**	10 (8)	23 (14)	0.13	27 (16)	7 (5)	0.01
**Home discharge from MTC (%)**	25 (20)	21 (13)	0.10	28 (17)	18 (14)	0.52

Data presented as median (IQR) unless otherwise indicated. TBI: Traumatic Brain Injury; BMI: Body Mass Index; ISS: Injury Severity Score; SBP: Systolic Blood Pressure; GCS: Glasgow Coma Scale; BD: Base Deficit; CSL:

Crystalloid; RBC: Red Blood Cells; IR: Interventional Radiology; CCLOS: Critical care length of stay; HLOS: Hospital length of stay; EoL: End of Life Care; MTC: Major Trauma Centre. ~survivors only. *Frail status unknown in 6 patients. p-value indicates comparison between not frail and frail, and no TBI and TBI groups (Mann Whitney U test, Chi Squared or Fishers Exact test).
